# Staying informed without a cost: No effect of positive news media on stress reactivity, memory and affect in young adults

**DOI:** 10.1371/journal.pone.0259094

**Published:** 2021-10-28

**Authors:** Charlotte Longpré, Claudia Sauvageau, Rebecca Cernik, Audrey-Ann Journault, Marie-France Marin, Sonia Lupien

**Affiliations:** 1 Centre for Studies on Human Stress, Institut Universitaire en santé mentale de Montréal, Research Center, CIUSSS Est-de-l’Île-de-Montréal, Montreal, Quebec, Canada; 2 Department of Psychology, Université de Montréal, Montreal, Quebec, Canada; 3 Department of Psychiatry and Addiction, Université de Montréal, Montreal, Quebec, Canada; 4 Department of Psychology, Université du Québec à Montréal, Montreal, Quebec, Canada; Ryerson University, CANADA

## Abstract

**Introduction:**

We read, see and hear news from various media sources every day. A large majority of the news is negative. A previous study from our laboratory showed that reading negative news is associated with both increased stress reactivity (measured via the stress hormone cortisol) and recall of the negative news segments in women.

**Objectives:**

The present study investigated the effects of positive news on cortisol stress reactivity, memory and affect using a methodology highly similar to the study on negative news that was previously used by our team.

**Methods:**

Sixty-two healthy participants aged between 18 and 35 years (81% women) were randomly exposed to either positive or neutral news segments, followed by a laboratory stressor. We assessed participants’ affect three times during the procedure and measured cortisol in saliva eight times (at 10-minute intervals). Twenty-four hours later, participants were contacted by phone to assess their recall of the news segments.

**Results:**

Results showed that exposure to positive news, relative to neutral news, did not modulate participants’ cortisol levels in response to the laboratory stressor. Positive news had no impact on memory recall of the news and did not change participants’ positive or negative affect. Bayes factors suggested that these nonsignificant results are not attributable to low statistical power.

**Conclusion:**

Contrary to negative news, positive and neutral news do not modulate stress reactivity, memory and affect. These results suggest that people can stay informed without physiological and psychological costs when the news to which they are exposed adopt a positive or neutral approach.

## Introduction

In its many diverse forms, media (social networks, newspapers, television) is ubiquitous in today’s society. In 2020, 43% of Canadians were still getting the news from printed newspapers. A large majority, however, read the weekly news via digital devices [1]. An analysis of the New York Times spanning from 1945 to 2005 [2] showed a significant increase in the trend for newspapers to present negative news. Consumers are aware of this trend as one report showed that 77% of Americans considered news to be negative and 84% considered news to be depressing [3].

Lazarus and Folkman [4] suggested that cognitive appraisal of a situation or an event as being negative activates the physiological stress system. When the brain perceives a threat, the hypothalamic-pituitary-adrenal axis is activated, leading to the secretion of cortisol by the adrenal glands [5]. This physiological reaction in response to a stressor is called reactive cortisol. Since cortisol is liposoluble, it can easily cross the blood-brain-barrier to reach receptors located in different brain regions such as the hippocampus [6], amygdala [7] and frontal lobe [8]. This is particularly important because these brain structures are involved in cognitive functions related to memory and emotion. Based on this physiological process, studies have assessed the effects of negative news on markers of stress such as cortisol and the impact of negative news on memory and emotions.

Results of these studies showed that negative news coverage of major events, such as continued watching of news related to 9/11, was associated with higher self-reported psychological symptoms of stress [9]. Furthermore, it has been shown that exposure to the Boston Marathon bombings via media coverage was associated with higher self-reported stress compared to direct exposure to the event a week after the bombings [10]. To date, only two studies have investigated the impact of negative news on cortisol levels. The first study exposed participants to a daily news television program and found no change in cortisol levels after exposure to the program [11]. The second study was performed by our research group. Marin et al. [12] were interested in testing whether exposure to negative news (compared to neutral news) would alter physiological reactivity to stress and could increase memory of the news. They found that women who read negative news before exposure to a laboratory stressor presented an increased cortisol reactivity following the stressor compared to women exposed to neutral news [12]. Women exposed to negative news also presented a greater recall of the news assessed 24 hours later. These modulating effects on stress reactivity and memory for negative news were only observed in women. These findings suggest the presence of a memory enhancing effect of threatening (negative) information and are important because of the pervasiveness of negative news and its perception by consumers.

Exposure to negative news has also been shown to modulate affective states. One study reported that, compared to positive news, reading negative news increased negative affect and decreased positive affect [13]. Another group investigated the effect of daily news exposure on emotions and found that if the news was perceived as negative by the reader, it was associated with more negative affect and less positive affect [14].

In response to the pervasiveness of negative news in our society and their consequences on stress, memory and emotion in the general population, a recent movement has emerged in the field of journalism called constructive journalism. Constructive journalism has been defined as a form of journalism that strives to apply various principles of positive psychology in the media to create more productive and engaging news [13]. In order to include these principles, news can present a solution, a hero or use more positive than negative words within the article. Such an approach helps to offer optimistic solutions and alternatives to today’s world [15] and counterbalances the negative trend in mainstream media [16].

To date, only few studies have been performed on constructive journalism with positive news [15]. One study showed that reading positive news led to an increase in positive affect, compared to negative news [15]. In the same study, reading constructive news (with a solution to a problem) was associated with higher positive affect than reading news without a solution or an optimistic view. A second study was also conducted using constructive news (presenting a solution within the news) in adolescents. Adolescents who read constructive news presented less negative affect [17]. A recent thesis showed that constructive news generated greater engagement among readers, although the effect was small [18]. Taken together, these results suggest that positive news may have opposite effects to those shown in the literature with negative news.

### Importance of the work

The aim of the current study was to determine if exposure to positive news affects cortisol levels following a psychosocial stressor and whether it influences memory recall of the news and/or affect in young adults. We postulated that compared to individuals in a neutral news condition, individuals exposed to positive news would display a decrease in cortisol levels in response to a psychosocial stressor. As emotional words and images seem to be easily recalled [19–21], we postulated that positive news would have better recall than neutral news and would increase positive affect and decrease negative affect. To test these hypotheses, we used a highly similar experimental research design to the one used by Marin et al. [12] for their study on negative news. Our study followed that of Marin et al. by attempting to extend the results found in their study, to positive news. Specifically, we tested whether positive news had as much impact on stress and memory as negative news.

## Methods and materials

### Ethics statement and disclosure

This project was approved by the Research Ethics Board of the Institut universitaire en santé mentale de Montréal (project number: 2019–1847). All participants provided written informed consent before participating in the study. This project was preregistered on the Open Science Framework and is accessible via the following link [DOI: 10.17605/OSF.IO/6CDEY].

### Participants

Participants were recruited by Facebook posts on student academic association pages, our research laboratory’s page and via advertisements posted in some universities in Montreal, inviting them to participate in a study on stress and media. The initial recruitment aimed for 120 healthy French-speaking young adults aged between 18 to 35 years old. Due to the COVID-19 pandemic, recruitment and testing were stopped in March 2020. As the pandemic represented an environmental stressor for the whole population, and because the media was very present throughout the crisis, resumption of the study was not possible. This would have created a bias between participants recruited before and after the pandemic. As such, 70 participants were recruited for this project between September 2019 and March 2020. Inclusion criteria included no psychiatric or physical (neurological, cardiovascular) disorder(s), being a non-smoker and no use of medication(s). Participants with a body mass index greater than 31 kg/m^2^ were excluded as obesity has been associated with elevated cortisol levels [22, 23]. Naturally cycling women, regardless of their menstrual phase as well as women using hormonal contraceptives, were included in the study in order to facilitate the comparison with Marin et al. that used the same inclusion criteria [12]. For a comparative table between the Marin et al. study and ours, please see Table 1 below. The menstrual cycle phase was determined according to the length of the cycle and the date of the last menstrual period for each naturally cycling woman. Screening by phone interview led to the exclusion of eight participants for medical reasons. The final sample included 62 participants (50 women). We are aware of the differential distribution of men and women in our sample and this factor has been considered in the analyses.

**Table 1:** Comparative table between Marin et al. (2012) and the current study.

	Marin et al. (2012)	Longpré et al. (2021)
Relevant information		
N	56 (28 women)	62 (50 women)
Number of news segments	24	12
News segments format	Title, first sentences of the news	Title, first sentences of the news
Time indexes and news selected occurred within a month	Yes	No
News type	Local news	Local and international news

This table highlights some similarities and differences between Marin et al. (2012) and the current study methodologies.

### Measures

#### Task design

We randomly assigned participants to one of two experimental conditions: a series of either positive or neutral news segments. Positive news segments were taken from GlobalGoodness, a website exclusively dedicated to publishing positive news from around the world. For instance, positive new segments surrounded topics such as vaccines, cancer treatment, and encouraging news about the environment. For neutral news, segments were taken from francophone newspapers in Montreal (La Presse, Le Journal de Montréal, Le Journal Métro). Examples of neutral news consisted of random facts on cats, news about TV shows or new album releases. One individual on the research team (CL) was responsible for the extraction of all news segments. Following the selection of all segments, we validated them to assess their valence (positive or neutral) to determine if the judgement made by the researcher was correct (see task validation in Results section). The final set of segments consisted of 24 news segments (12 positive and 12 neutral) unlike Marin et al. [12], please refer to Table 1. We did not use the same number of segments as Marin et al. [12] due to the lack of recent positive news available in the media. Indeed, even if *GlobalGoodness* is dedicated to the production of positive news, the small number of positive news published did not allow us to find a large number of recent segments. Consequently, from the 12 positive and 12 neutral news segments collected, we removed all time indexes. All segments included the title, first lines of the article and were presented as visually written segments (to read the selected segments, refer to S1 Table). This was done to control for the potential impact of double stimulation on memory, which can be induced when both a text and an image are presented together [24, 25]. The task was programmed and administered via Qualtrics, a highly secure web-based platform [26]. In each group, we told participants that news segments would be presented randomly on the screen and that each segment would last for 25 seconds. After 25 seconds, participants were instructed to click to read the next news segment. We did not collect any information on whether participants were exposed to the selected news segments prior their laboratory visit (either positive or neutral).

We assessed news recall via a phone call 24 hours after participants’ exposure to the news segments. Participants were unaware that they would have to recall the news 24 hours later. During the phone call, participants were asked to recall as many news segments (that they had read the previous day) in as much detail as possible. The interviewer transcribed the call for two judges to rate thereafter. Both judges needed to be able to identify the news segment described by the participant to attribute a point (a score of 1 for each correctly identified news segment). The experimenter then read each of the news segment to the participant. Using a Likert-type scale, the participant had to evaluate the emotionality of each news segment (1 being neutral/not positive and 5 being truly positive). The participant was also asked to assess how concerned he/she felt by the news (1 being unconcerned/slightly concerned and 5 being very concerned).

#### Psychosocial stressor

The stressor used for this study was the Trier Social Stress Test (TSST), a highly validated psychosocial stressor known to increase cortisol levels [27]. In brief, the TSST consisted of a 10-minute anticipation period followed by a 10-minute testing period. The testing period was divided into two parts: the first part involved a 5-minute oral presentation where the participant was asked to engage in a job interview and the second part involved a 5-minute period of mental arithmetic. In line with Marin et al. [12] and other previous studies from our laboratory [28, 29], we used the panel-out variant of the TSST. With this variant, participants faced a false-mirror and a judge (another research team member) communicated with them via a microphone. The judge was presented to the participants as a behavioral expert. Besides this difference, the protocol was the same as the original variant of the TSST [27].

#### Cognitive tasks

To ensure that participants did not suffer from any memory impairment that would impact their ability to recall the news, they completed a logic memory task from the Weschler Memory Scale 3^rd^ edition [30] as a control task. In summary, the task involved an experimenter who read a short 90-word text to the participant. An immediate recall was performed directly after the reading and a delayed recall was done 20 minutes later. Another task was designed to ensure that participants had sufficient basic French reading comprehension skills and could understand the news segments used in the main task. After the participant quietly read a text, the experimenter asked him/her five comprehension questions. A mean score was then computed to determine if the participant had a satisfactory understanding of the text.

#### Questionnaire

To measure the impact of positive news on affect, we used the Positive and Negative Affect Scale (PANAS) at three different time points during the protocol [31]. This scale assesses emotional state on two dimensions: positive affect and negative affect. For both scales, scores range from 10 to 50 where a higher score represents higher levels of positive/negative affect. According to the literature, the reliability coefficient of the Positive Affect scale is 0.89 and 0.85 for the Negative Affect scale. In the present sample, Cronbach’s alpha for the Positive Affect Scale was 0.85 and 0.74 for the Negative Affect Scale. Participants also completed additional questionnaires in this study, though they are not discussed in this paper. However, in line with the transparency of reporting checklist guidelines [32], a complete list of tasks and questionnaires used in this study can be found on the Open Science Framework platform at DOI 10.17605/OSF.IO/UZYRC [PROTOCOL DOI]. All questionnaires were completed in the laboratory on a computer using Qualtrics [26].

#### Salivary cortisol assays

Cortisol levels were measured in saliva. Saliva samples were kept at -20°C until the analyses were performed. Samples were processed at the salivary analysis laboratory, of the Centre for Studies on Human Stress, using high-sensitivity immunological enzyme dosing from Salimetrics State College, catalogue number 1–3102. The degree of detection of this analysis is 0.012–3 ug/dL. The cortisol extraction protocol is described in detail elsewhere [33].

#### Procedure

**Fig 1:**
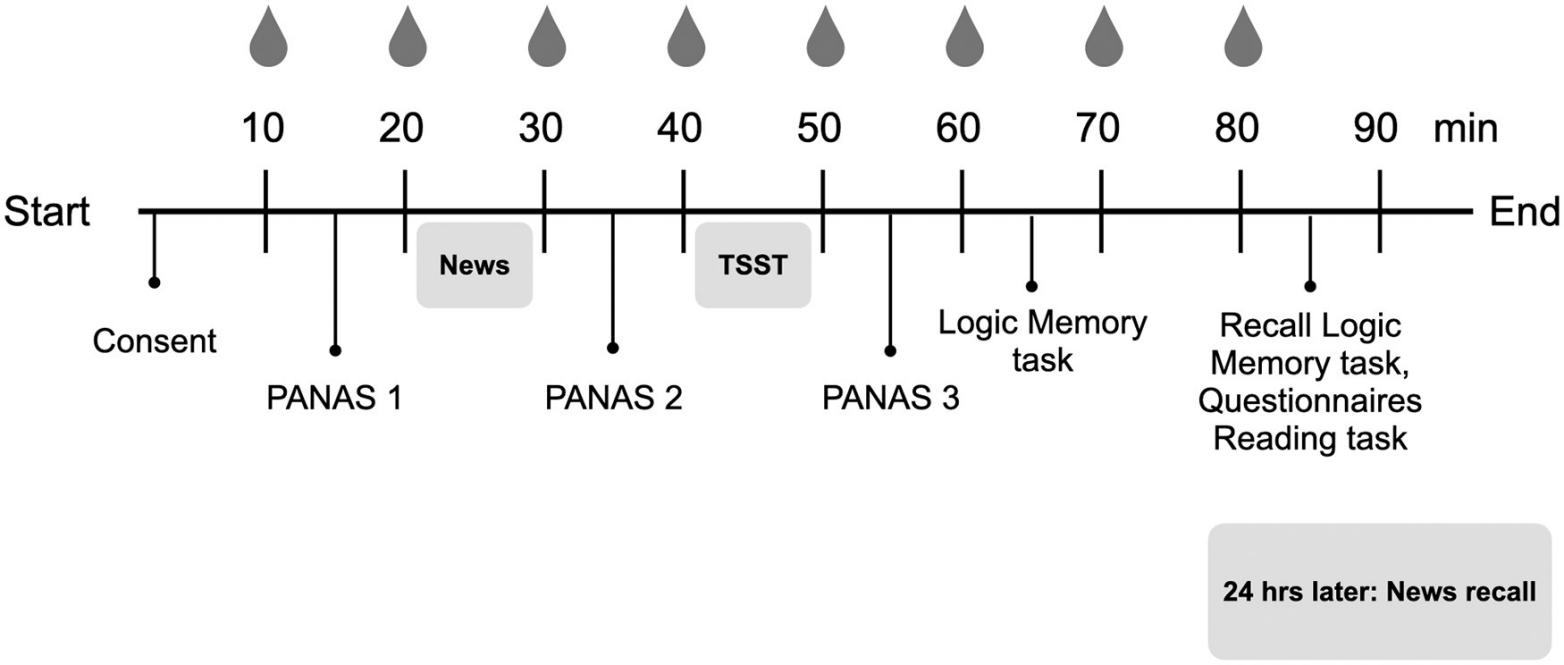
Schematic representation of the procedure. The figure shows the timeline for the laboratory visit.

Participants were scheduled for a 2-hour lab visit and a 15-minute phone interview 24 hours later. To control for circadian cortisol levels, all laboratory visits occurred between 12:00 PM and 4:30 PM. During their lab visit, participants completed the PANAS before and after reading the news segments (positive or neutral news depending on their condition). They were then exposed to the TSST and completed the PANAS one last time. Finally, participants performed the logic memory task and the reading task. We collected a total of eight saliva samples, where samples were taken at 10-minute intervals throughout the entire laboratory visit (Fig 1). Participants’ recall of the news segments was tested during a phone interview 24 hours later.

## Statistical analyses

### Preregistration

All statistical analyses were preregistered on the Open Science Framework platform [DOI 10.17605/OSF.IO/6CDEY] and data are available at DOI 10.17605/OSF.IO/4ZX98 [34]. Analyses were performed using Statistical Package for Social Science (SPSS) version 26 [35]. It was initially planned to perform all analyses using sex as an independent variable. However, given premature study cessation due to the COVID-19 pandemic and the unequal distribution of men and women within the sample, sex differences were not examined. However, sex was included as a covariate in all analyses. This resulted in a modification to the type of analyses to be conducted, compared to the analyses we originally preregistered.

### Data cleaning

Data cleaning was performed using R software [36]. We checked for extreme values for TSST cortisol measurements. To do so, measurements with z- scores exceeding the cut-off value of ± 3.29 were considered extreme. Extreme data were transformed via winsorization. For a cut-off winsorization of z = ± 3.29, all observed values below the 0.1 percentile were replaced with a z-score corresponding to a value of -3.29 and all observed values above the 99.9 percentile were replaced with a z-score of +3.29. Data for one participant was replaced using this technique. Upon discovery that the cortisol data was not normally distributed, a log-transformation was performed on raw cortisol data before conducting all analyses. However, for the purpose of reporting valid endocrine data, figures represent untransformed cortisol data. Raw data were used for the analysis of psychological measures (news recall and other questionnaires).

### Preliminary analyses

#### Task validation

First, it was necessary to validate the experimental task by ensuring that the selected news segments for the positive condition were evaluated by the participants as being more positive than the neutral news segments. One-way ANCOVAs were performed on the scores obtained for Emotionality and Concerned (for each news segment), with Condition (neutral or positive news) as the between-subjects factor and Sex as covariate.

#### Cognitive skills

To ensure that participants in both conditions did not suffer from any memory impairment and possessed sufficient reading abilities, t-tests (two-tailed) were performed using the scores obtained on the logic memory task and reading task.

### Main analyses

#### Objective 1

To determine if exposure to positive news segments led to changes in cortisol levels in response to the TSST, a repeated measures ANCOVA with Time (time point of collected saliva sample) as the within-subjects factor and Condition (neutral, positive) as the between-subjects factor was conducted on cortisol values. Considering the potential effect of sex hormones on cortisol levels [37], we controlled for sex hormones (and the use of hormonal contraceptive). To do so, we created a variable called Sex hormones (with four levels: men, luteal phase, follicular phase and use of hormonal contraceptives). This variable was used as a covariate in the analysis.

#### Objective 2

To investigate the potential impact of exposure to positive news on memory recall, a one-way ANCOVA with Condition as the between-subjects factor was conducted using the total score for news segments remembered 24 hours later. Here, we only controlled for Sex.

#### Objective 3

To determine if exposure to positive news segments increased positive affect and/or decreased negative affect, two repeated measures ANCOVAs were performed using Time (PANAS measured at three different time points) as the within-subjects factor and Condition as the between-subjects factor, with the score obtained on the PANAS for Positive Affect and Negative Affect. Again, Sex was used as a covariate.

## Results

Sociodemographic characteristics of the sample are presented in Table 2. Most of the participants were White university students with a mean age of 23.9 years (*SD* = 4.4). Although assignment to experimental conditions was randomized in the study, we found a significant difference of ethnicity between conditions (greater number of White participants in the neutral news condition) *X*^*2*^ (6, *N* = 62) = 13.411, *p* = 0.037. To further investigate this difference, we conducted our main analyses by including Ethnicity as a covariate. It was later found to have no impact on the results (which are presented below). In addition, we performed a one-way ANOVA on the total score of news segments remembered with Ethnicity as a between-subjects factor. We found that the group difference was nonsignificant (*F*(6,55) = 0.514, *p* = 0.795). Consequently, despite an observed difference of ethnicity between the conditions, it did not appear to impact our results and was not included in subsequent analyses.

**Table 2:** Sociodemographic participant’s information.

	Neutral news		Positive news		Total sample	
Sociodemographic information	n	%	n	%	n	%
Group	30	48.4	32	51.6	62	
Sex^a^						
Female	26	86.7	24	75	50	80.6
Male	4	13.3	8	25	12	19.4
Menstrual phase						
Follicular	1	3.3	3	9.4	4	6.5
Luteal	8	26.7	7	21.9	15	24.2
Hormonal contraceptives	15	50.0	9	28.1	24	38.7
Missing	2	6.7	5	15.6	7	11.3
Socioeconomic Status						
$0 –$18,012 (poverty line)	16	53.3	21	65.6	37	59.7
$18,012 –$25,000	3	10.0	4	12.5	7	11.3
$25,000 –$50,000	4	13.3	3	9.4	7	11.3
$50,000 –$75,000	3	10.0	4	12.5	7	11.3
$75,000 –$100,000	1	3.3	0	0	1	1.6
Missing	3	10.0	0	0	3	4.8
Civil Status						
Single	15	50.0	19	59.4	34	54.8
Cohabiting	10	33.3	8	25.0	18	29.0
Married/partnered	5	16.7	5	15.7	10	16.1
Employment						
Student^b^	21	70.0	27	84.4	48	77.4
Employed^b^	11	36.7	9	28.1	20	32.3
Ethnicity						
White	23	76.7	15	46.9	38	61.3
Chinese	2	6.7	0	0.0	4	6.4
South-East Asian	0	0.0	1	3.1		
West Asian	0	0.0	1	3.1		
Black	4	13.3	5	15.6	9	14.5
Arabic	1	3.3	5	15.6	6	9.7
Latin American	0	0.0	5	15.6	5	8.1

^a^The unequal sex distribution is discussed further.

^b^Participants answered “yes” to the question.

### Preliminary analyses

**Fig 2:**
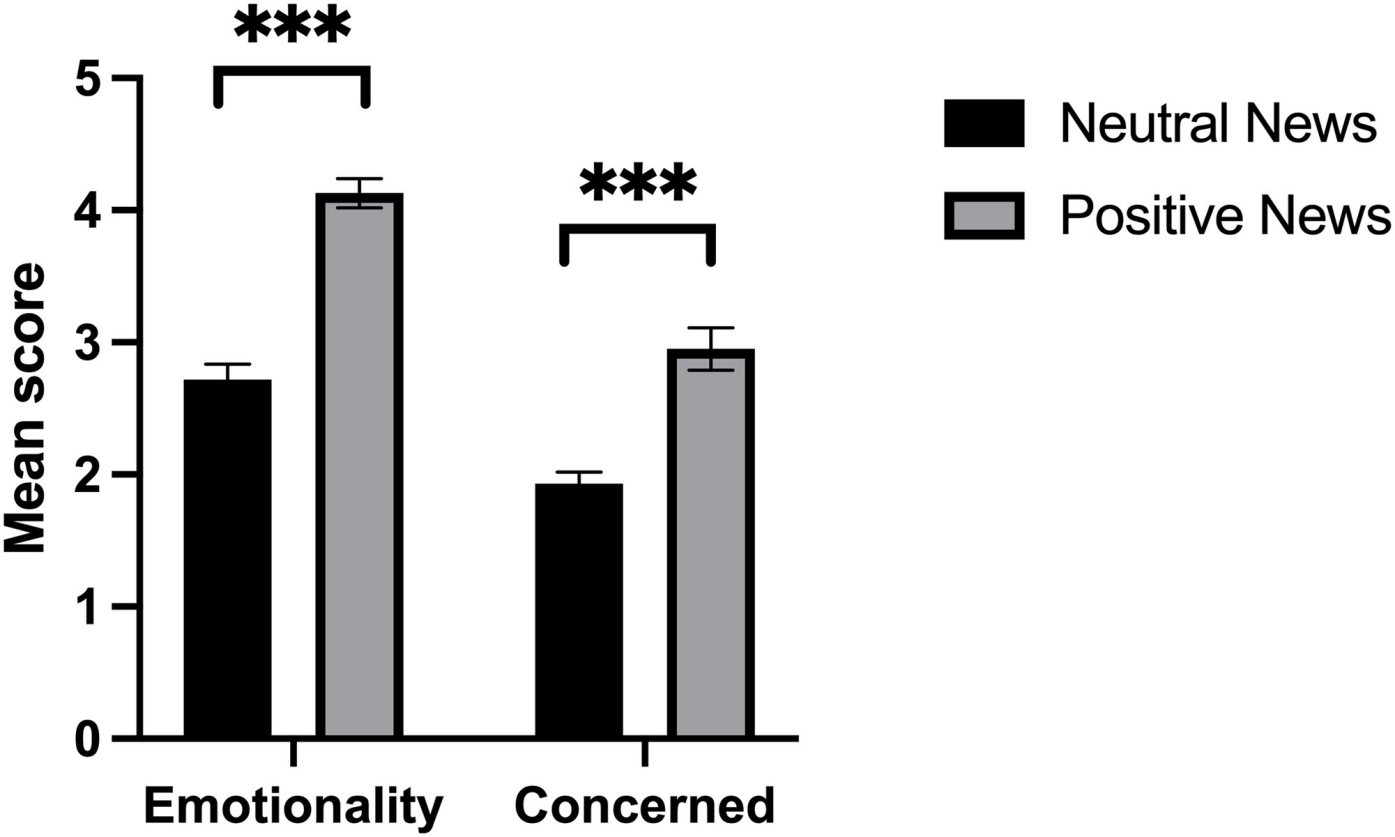
Mean score on emotionality and concerned about the news as a function of condition. Non-adjusted means are presented and errors bars are the standard error of the mean. ***p<0.001.

Regarding task validation, one-way ANCOVAs revealed a significant effect of Condition for Emotionality (*F*(1,58) = 90.077, *p* < 0.001; d = 2.28) and Concerned (*F*(1,58) = 33.418, *p* < 0.001; d = 1.41) confirming that the positive news condition was associated with higher scores for Emotionality and Concerned (Fig 2).

Regarding cognitive skills, ANCOVAs revealed that there was no significant difference between conditions for the logic memory task (*F*(1,59) = 0.051, *p* = 0.822) and the reading task (*F*(1,59) = 0.864, *p* = 0.356).

### Main analyses

**Fig 3:**
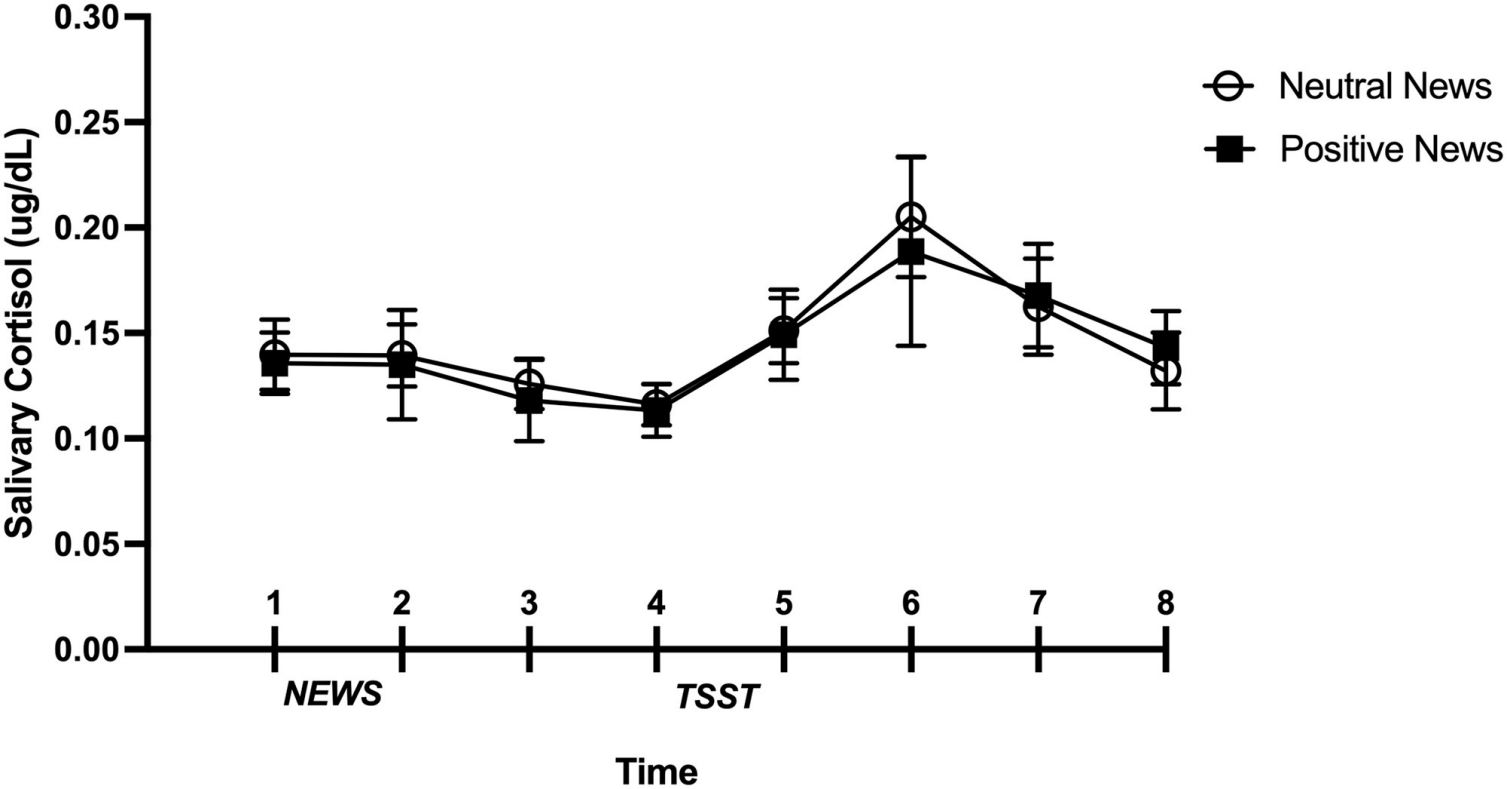
Salivary cortisol levels in response to news reading and TSST as a function of condition. Non-adjusted means of raw cortisol data were used in this graph and standard errors of the means were used for the error bars.

For the repeated measures ANCOVAs performed on salivary cortisol levels, the assumption of sphericity was violated and consequently, an average of the Greenhouse-Geiser and Huynh-Feldt corrections was used [38]. The analysis revealed no significant main effect of Time (*F*(2.26, 113.27) = 2.14, *p* = 0.12, n_p_^2^ = 0.041), Condition (*F*(1,50) = 0.05, *p* = 0.82, n_p_^2^ = 0.001), nor the Condition by Time interaction for salivary cortisol levels (*F*(2.26, 113.27) = 0.09, *p* = 0.93, n_p_^2^ = 0.002). Thus, participants who read positive news presented the same cortisol reactivity to the TSST as participants who read neutral news (Fig 3).

**Fig 4:**
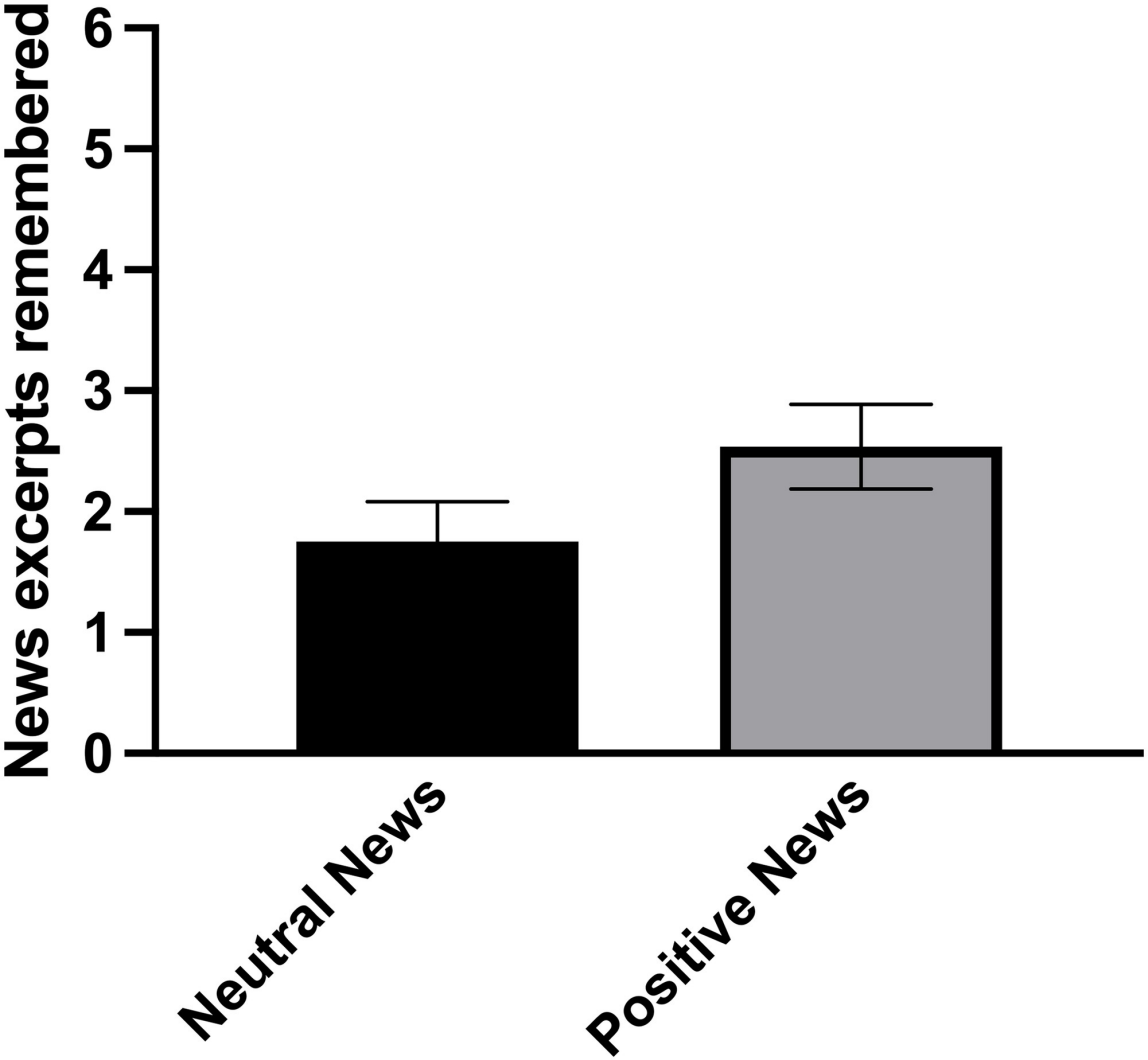
Average number of news segments remembered as a function of condition. Standard errors of the means were used for the error bars.

The one-way ANCOVA testing the impact of positive news on memory recall indicated no significant difference between conditions (*F*(1,59) = 2.168, *p* = 0.146, n_p_^2^ = 0.035; Fig 4), showing that participants who read positive news remembered as many news segments as participants who read neutral news.

**Fig 5:**
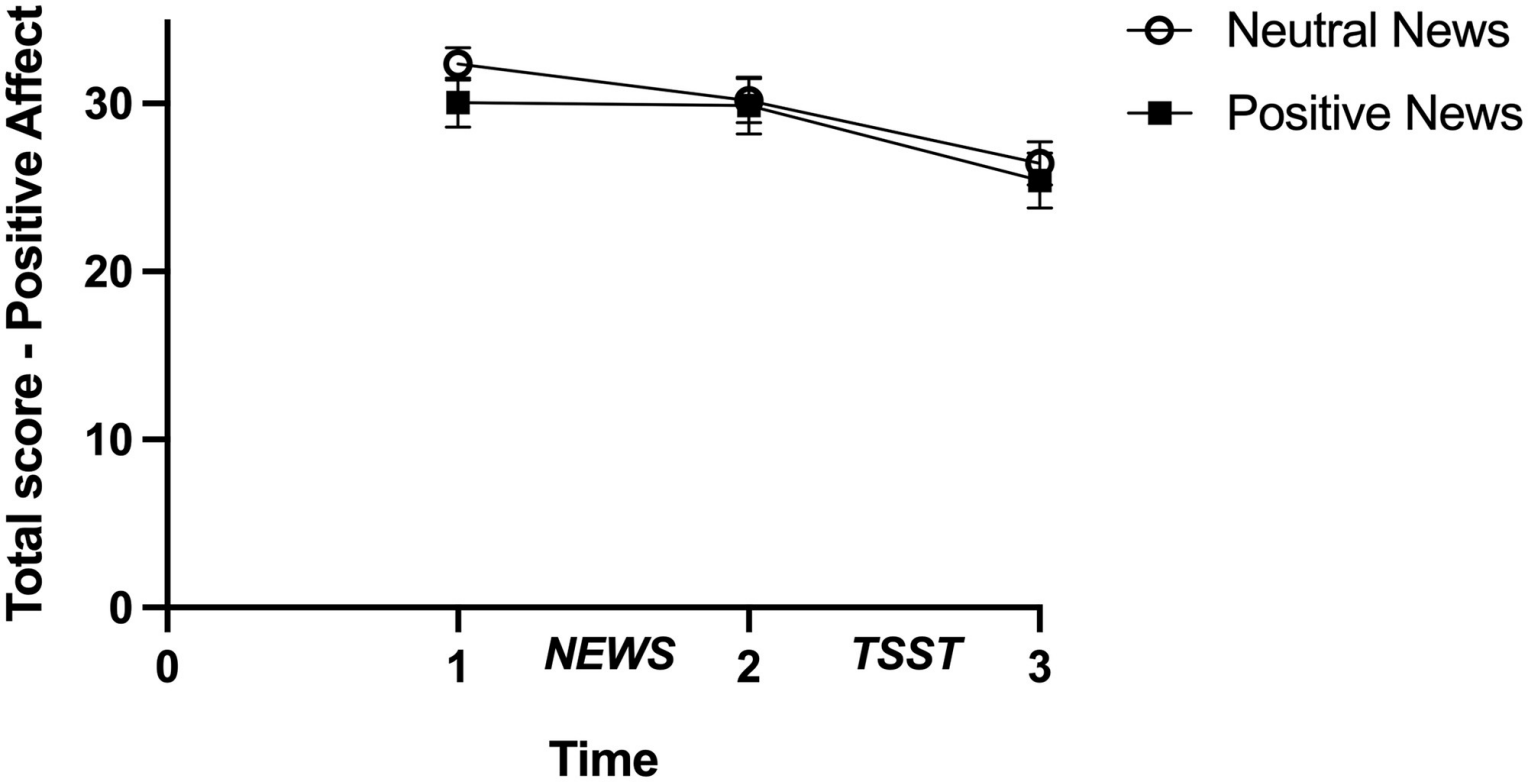
Score on positive affect, PANAS’s subscale in function of time as a function of condition. Non-adjusted means are presented and errors bars are the standard error of the mean.

**Fig 6:**
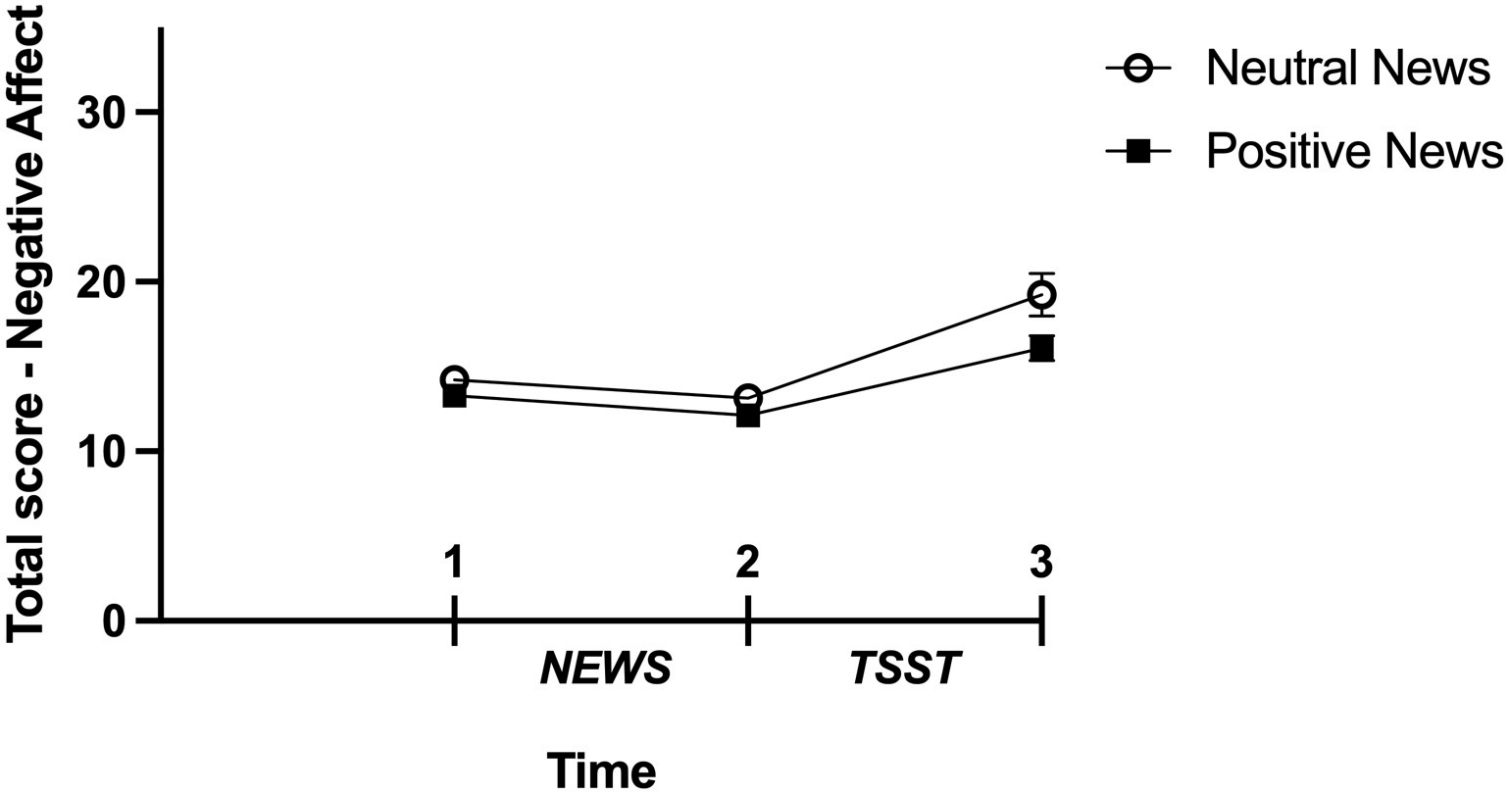
Score on negative affect, PANAS’s subscale in function of time as a function of condition. Non-adjusted means are presented and errors bars are the standard error of the mean.

Due to violation of the assumption of sphericity, the average of the Greenhouse-Geiser and Huynh-Feldt corrections were used [38] for the analysis regarding change in affect. The analysis revealed a significant main effect of Time for positive affect (*F*(1.57, 92.81) = 33.47, *p* < 0.001, n_p_^2^ = 0.362) and negative affect (*F*(1.25, 73.95) = 34.23 *p* < 0.001, n_p_^2^ = 0.367) showing that as time went by, positive affect decreased and negative affect increased. However, results were nonsignificant for the main effect of Condition for positive (*F*(1,59) = 0.50, *p* = 0.483, n_p_^2^ = 0.008) and negative affect (*F*(1, 59) = 3.22, *p* = 0.078, n_p_^2^ = 0.052) and for the Condition by Time interaction for positive (*F*(1.57, 92.81) = 1.21, *p* = 0.295, n_p_^2^ = 0.020) and negative affect (*F*(1.25, 73.95) = 2.63, *p* = 0.101, n_p_^2^ = 0.043; Figs 5 and 6).

### Evidential value of null results using bayes factors

Considering the nonsignificant results found in the main analyses, we used Bayes factors to assess their evidential values. According to Aczel et al. [39, 40], null results can occur for two main reasons. Either there is a lack of power to detect the effect, or the effect tested does not exist. To test these two possibilities, we used the Bayes information criterion (BIC), a ratio that assesses the likelihood of the null hypothesis (H_0_ = no difference between groups) compared to the likelihood of the alternative hypothesis (H_1_ = there is a significant difference [39, 40].

To further investigate the absence of differences between conditions on cortisol levels in response to the TSST, we calculated BIC using JASP software [41]. The BIC for the difference in cortisol levels between conditions was 60.92. As proposed by Jarosz and Wiley [42], this value represents a strong evidence in favor of the null hypothesis (no difference in cortisol levels between conditions), suggesting that the lack of power does not explain the absence of a difference in cortisol levels between conditions. Considering that no difference was found on memory recall between conditions, we also computed BIC. The BIC for this result was 7.05, representing substantial evidence in favor of H_0_ (there is no difference on memory recall between conditions), suggesting that the null result cannot be explained by a lack of statistical power [42]. However, this evidential value is weaker than the one obtained for cortisol levels. Finally, we computed BIC for the absence of difference between conditions on the PANAS. The BIC for the Positive Affect Scale was 3.80, indicating substantial evidence in favor of H_0_ (no difference on positive affect between conditions) and 2.40 for the Negative Affect Scale, indicating anecdotal difference in favor of H_0_. This suggests that we may have failed to detect a difference in negative affect between participants exposed to positive and neutral news due to a lack of statistical power.

## Discussion

The three main objectives of this study were to determine whether exposure to positive news segments, compared to exposure to neutral news segments, would lead to lower stress reactivity to a laboratory stress, a greater recall of news 24 hours later, an increase in positive affect and a decrease in negative affect. Our findings did not support our hypotheses, as no group differences were observed for either of these measures.

Contrary to our hypothesis, exposure to the positive news segments did not lead to lower cortisol reactivity to the laboratory stressor but at the same time, it did not lead to an increase in cortisol reactivity as reported by Marin et al. [12] with negative news. This result suggests that positive or neutral news can serve to inform individuals without a physiological cost. In this sense, exposure to positive news could be a way to stay informed without increased stress reactivity as observed with negative news [12]. This suggestion aligns with studies showing that humans are more attracted and reactive to negative content [43–45]. This phenomenon is called the negativity bias and represents a natural human tendency, as well as serving an evolutionarily purpose for threat detection in the environment [46]. As the brain’s principal task is to ensure survival, this implies that negative content will always appear more salient and will be detected more rapidly than positive or neutral information [44]. The negativity bias is present in multiple domains [46, 47] and has been marginally studied in the news [48–51]. Consequently, a possible explanation for the absence of a decrease in stress reactivity may lie in the fact that the positive news in the current study were not positive enough to counterbalance the brain’s natural tendency to detect threat. It is also possible that the number of news segments presented was not sufficient to prevent the negativity bias and reduce stress reactivity. Only 12 news segments were selected for this study due to the lack of positive news in the media. This might have prevented the development of a cumulative effect of positive news and therefore a decreasing effect on stress reactivity. Further studies should increase the number of positive news segments to which individuals are exposed and increase the positivity of the news to determine whether exposure to positive news can eventually lead to a significant decrease in stress reactivity and increase memory of positive aspects of the news.

Based on data provided by the emotion and memory literature [19–21], we postulated that positive news would be better recalled than neutral news. Our results showed that compared to neutral news, exposure to positive news segments before a social stressor did not improve news recall in young healthy adults. Given that cortisol can be a facilitator for encoding information [20], the absence of a difference in cortisol levels between the two conditions may explain why memory recall did not differ for participants exposed to either positive or neutral news. In addition, it is possible that a greater number of news segments would lead to a better recall. It is thus possible that there exists a threshold of activation at which the brain will respond to positive news and modulate the biological stress response and concomitant memory. Another possibility for the absence of a memory effect for positive news is the fact that all news segments used in the current study lacked time indexes. As it was impossible to include only recent positive news, all time indexes were removed from the segments. As time can be used to provide context during encoding [52, 53], future studies should include temporal information in positive news segments to investigate whether the amount of time elapsed since the event impacts news recall.

Even though the positive news segments were considered more positive than the neutral segments and that participants felt more concerned by them, positive news did not affect positive and negative affect in participants. However, a study in constructive journalism showed that exposure to positive news increased positive affect and decreased negative affect [15]. We were unable to replicate these results in our study. The inability to induce positive affect or decrease negative affect may be explained by the presence of the stressor. Exposure to a stressor typically induces negative emotions [54] and thus a stress-induced negative emotion may have prevented any affect-enhancing effect of positive news. Moreover, the current study used news segments (short segments of whole news articles, the title and the first sentences). Yet, it is possible that the positive news segments in the current study lacked context to induce positive emotions which may have resulted in no affect change. To determine if it is possible to replicate results derived from constructive journalism [15], future studies could include the entire news piece instead of only short segments. Finally, due to the international nature of the *GlobalGoodness* platform, it is possible that people did not feel geographically close enough to the location of the news to generate positive affect. Therefore, it would be interesting to study the impact of local positive news on affect in future studies.

The lack of difference in stress reactivity, memory and affect between participants exposed to positive and neutral news was supported by the calculation of a Bayes factor. The interpretation of the Bayes factor computed for our main analyses (cortisol, memory recall and positive affect) suggested that the lack of difference cannot be explained by a lack of statistical power [42]. However, the BIC value obtained for the difference in negative affect was 2.40 (anecdotal difference in favor of H0). It is thus possible that we may have failed to detect a difference in negative affect between the positive and the neutral news conditions. We used the BIC instead of interpreting nonsignificant p-values mainly because of the difficulties in interpreting p values [55]. Yet, it is important to note that Bayes factors still rely on cut-offs and that the conclusions of our analyses may be affected by these cut-offs.

This study was unique as it was the first study to use an experimental design and cortisol sampling with real positive news segments. This study contributes to the literature and allows to generate new hypotheses for future research. However, our study was not without limitations. First, due to COVID-19, we were unable to recruit a large number of participants and our final sample had a large discrepancy between men and women. This limits the generalization of our results to men and general population as our sample was predominantly composed of White undergraduate students. Second, and as previously mentioned, the use of short news segments and lack of positive news within the media prevented us from using recent news as stimuli. Third, participants may have been exposed to positive news or neutral news segments used in the current study through the media (while reading the newspaper) prior to their participation in the study. Unfortunately, we did not control for this variable. Finally, the ecological validity of this study may have been limited as this study was conducted in a laboratory setting and may not be representative of real-world experiences (i.e., reading the news undisturbed and without being exposed to a laboratory stressor). Therefore, we propose that future studies should be done in a more ecological context. For example, it would be interesting to test the impact of receiving positive news (e.g., a job promotion) before a stressor (e.g., an important meeting). In that context, we may expect that positive news would buffer the perceived threat (meeting) and consequently the stress response associated to this threatening event. Future studies should test this potential buffering effect of positive news prior to a stressor in a real-life context where participants would report any positive and negative news received during the day.

In the current study, we specifically tested the effect of reading positive news on the global stress response to the TSST. We investigated the difference between groups on the stress response after exposure to positive news or neutral news. More specifically, we wanted to test the buffer (or inoculation) effect of positive news to a subsequent stressor. However, it is possible that a recovery effect exists, and it should be pertinent to conduct future studies to test this. The possible recovery effect of positive news would appear if reading positive news after exposure to a stressor would lead to a faster recovery to baseline cortisol levels following the stress. These more specific and distinct hypotheses on the role of positive news on stress reactivity are relevant and could be tested in future research.

In brief, our study highlights that positive news does not affect individuals’ cortisol levels, memory or affect. Compared to negative news, reading positive news did not lead to increased stress reactivity, nor did it heighten memory [12], increase negative affect or decrease positive affect [15]. In other words, exposure to positive news could allow people to stay informed about the latest news without the cost of negative news on physiological and/or psychological processes. Beyond this, it is possible that exposure to positive news subsequent to negative news exposure could lead to a faster recovery from the stress induced by the negative news. If this hypothesis is confirmed in future studies, this would suggest that constructive or positive news could be a way to offset the physiological and psychological changes induced by negative news.

## Supporting information

S1 Table News segments (neutral news and positive news) used in the current study. 12 neutral news and 12 positive news segments used as stimuli in the present study. The items have been translated into English for the purpose of this article, but the original language of the news is French.
(PDF)

## References

[1] Weekly newspaper reach in Canada in March 2019 and November 2020, by platform [Internet]. Statista. 2021 [cited 2021 Aug 6]. https://www.statista.com/statistics/261807/reach-of-select-media-in-canada/

[2] Leetaru K. Culturomics 2.0: Forecasting large-scale human behavior using global news media tone in time and space. First Monday [Internet]. 2011 Aug 17 [cited 2020 Oct 26]; https://firstmonday.org/ojs/index.php/fm/article/view/3663

[3] Patterson TE. Doing Well and Doing Good: How Soft News and Critical Journalism Are Shrinking the News Audience and Weakening Democracy–And What News Outlets Can Do About It. Harvard Unversity; 2000 p. 28.

[4] Lazarus R, Folkman S. Stress appraisal and coping. New York: Springer Publishing Company; 1984. 456 p.

[5] Sapolsky RM, Romero LM, Munck AU. How Do Glucocorticoids Influence Stress Responses? Integrating Permissive, Suppressive, Stimulatory, and Preparative Actions. Endocr. Rev. 2000;21(1):55–89. doi: 10.1210/edrv.21.1.0389 10696570

[6] Herman JP, Ostrander MM, Mueller NK, Figueiredo H. Limbic system mechanisms of stress regulation: Hypothalamo-pituitary-adrenocortical axis. Prog Neuropsychopharmacol Biol Psychiatry. 2005 Dec;29(8):1201–13. doi: 10.1016/j.pnpbp.2005.08.006 16271821

[7] van Stegeren AH, Wolf OT, Kindt M. Salivary alpha amylase and cortisol responses to different stress tasks: Impact of sex. Int J Psychophysiol. 2008 Jul;69(1):33–40. doi: 10.1016/j.ijpsycho.2008.02.008 18417235

[8] Diorio D, Viau V, Meaney M. The role of the medial prefrontal cortex (cingulate gyrus) in the regulation of hypothalamic-pituitary-adrenal responses to stress. J Neurosci. 1993 Sep 1;13(9):3839–3847. doi: 10.1523/JNEUROSCI.13-09-03839.1993 8396170 PMC6576467

[9] Schuster MA, Stein BD, Jaycox LH, Collins RL, Marshall GN, Elliott MN, et al. A National Survey of Stress Reactions after the September 11, 2001, Terrorist Attacks. N Engl J Med. 2001 Nov 15;345(20):1507–1512. doi: 10.1056/NEJM200111153452024 11794216

[10] Holman EA, Garfin DR, Silver RC. Media’s role in broadcasting acute stress following the Boston Marathon bombings. Proc Natl Acad Sci. 2014 Jan 7;111(1):93–98. doi: 10.1073/pnas.1316265110 24324161 PMC3890785

[11] Ragonesi AJ, Antick JR. Physiological Responses to Violence Reported in the News. Percept Mot Skills. 2008 Oct 1;107(2):383–395. doi: 10.2466/pms.107.2.383-395 19093600

[12] Marin M-F, Morin-Major J-K, Schramek TE, Beaupré A, Perna A, Juster R-P, et al. There Is No News Like Bad News: Women Are More Remembering and Stress Reactive after Reading Real Negative News than Men. Uddin M, editor. PLOS One. 2012 Oct 10;7(10):e47189. doi: 10.1371/journal.pone.0047189 23071755 PMC3468453

[13] Gyldensted C. Innovating News Journalism through Positive Psychology [Internet]. [Pennsylvania]: University of Pennsylvania; 2011. https://repository.upenn.edu/mapp_capstone/20/?utm_source=repository.upenn.edu%2Fmapp_capstone%2F20&utm_medium=PDF&utm_campaign=PDFCoverPages

[14] Hoog N, Verboon P. Is the news making us unhappy? The influence of daily news exposure on emotional states. Br J Psychol [Internet]. 2019 Mar 21 [cited 2020 Jan 24]; https://onlinelibrary.wiley.com/doi/abs/10.1111/bjop.12389 30900253 10.1111/bjop.12389 PMC7187375

[15] McIntyre KE. Constructive Journalism: The Effects of Positive Emotions and Solution Information in News Stories. [Chapel Hill]: University of North Carolina; 2015.

[16] McIntyre KE, Gyldensted C. Constructive Journalism: An Introduction and Practical Guide for Applying Positive Psychology Techniques to News Production. J Media Innov. 2017 Jan 28;4(2):20–34.

[17] Kleemans M, Schlindwein LF, Dohmen R. Preadolescents’ Emotional and Prosocial Responses to Negative TV News: Investigating the Beneficial Effects of Constructive Reporting and Peer Discussion. J Youth Adolesc. 2017;46(9):2060–2072. doi: 10.1007/s10964-017-0675-7 28424952 PMC5561152

[18] Kozminski-Martin A. L’effet du journalisme constructif sur l’engagement du lectorat d’un site Web d’information [Internet] [Mémoire]. [Montréal]: Université de Montréal; 2018. https://papyrus.bib.umontreal.ca/xmlui/handle/1866/20667

[19] Buchanan TW, Lovallo WR. Enhanced memory for emotional material following stress-level cortisol treatment in humans. Psychoneuroendocrinology. 2001 Apr;26(3):307–317. doi: 10.1016/s0306-4530(00)00058-5 11166493

[20] Cahill L, Gorski L, Le K. Enhanced Human Memory Consolidation With Post-Learning Stress: Interaction With the Degree of Arousal at Encoding. Learn Mem. 2003 Jul;10(4):270–274. doi: 10.1101/lm.62403 12888545 PMC202317

[21] Schwabe L, Bohringer A, Chatterjee M, Schachinger H. Effects of pre-learning stress on memory for neutral, positive and negative words: Different roles of cortisol and autonomic arousal. Neurobiol Learn Mem. 2008 Jul;90(1):44–53. doi: 10.1016/j.nlm.2008.02.002 18334304

[22] Mcewen BS. Protective and Damaging Effects of Stress Mediators. N Engl J Med. 1998;338(3):171–179. doi: 10.1056/NEJM199801153380307 9428819

[23] Defining Adult Overweight and Obesity | Overweight & Obesity | CDC [Internet]. 2020 [cited 2021 Feb 2]. https://www.cdc.gov/obesity/adult/defining.html

[24] Frieske DA, Park DC. Memory for news in young and old adults. Psychol Aging. 1999;14(1):90–98. doi: 10.1037//0882-7974.14.1.90 10224634

[25] Wingfield A, Stine EAL, Myers SD. Age Differences in Processing Information From Television News: The Effects of Bisensory Augmentation. J Gerontol. 1990 Jan 1;45(1):P1–P8. doi: 10.1093/geronj/45.1.p1 2295777

[26] Smith R, Smith SM, Orgill S, Smith J. Qualtrics—XM [Internet]. Washington: Qualtrics; 2002 [cited 2021 Jun 2]. https://www.qualtrics.com/fr/

[27] Kirschbaum C, Pirke K-M, Hellhammer DH. The ‘Trier Social Stress Test’—a tool for investigating psychobiological stress responses in a laboratory setting. Neuropsychobiology. 1993;28:76–81. doi: 10.1159/000119004 8255414

[28] Juster R-P, Sindi S, Marin M-F, Perna A, Hashemi A, Pruessner JC, et al. A clinical allostatic load index is associated with burnout symptoms and hypocortisolemic profiles in healthy workers. Psychoneuroendocrinology. 2011 Jul;36(6):797–805. doi: 10.1016/j.psyneuen.2010.11.001 21129851

[29] Raymond C, Marin M-F, Wolosianski V, Journault A-A, Longpré C, Leclaire S, et al. Early childhood adversity and HPA axis activity in adulthood:The importance of considering minimal age at exposure. Psychoneuroendocrinology. 2021;124(105042):1–9. doi: 10.1016/j.psyneuen.2020.105042 33249330

[30] Weschler D. WMS-III administration and scoring manual. 3rd ed. The Psychological Corporation; 1997b.

[31] Watson D, Clark LA, Tellegen A. Development and validation of brief measures of positive and negative affect: The PANAS scales. J Pers Soc Psychol. 1988;54(6):1063–1070. doi: 10.1037//0022-3514.54.6.1063 3397865

[32] Macleod M, Collings AM, Graf C, Kiermer V, Mellor D, Swaminathan S, et al. The MDAR (Materials Design Analysis Reporting) Framework for transparent reporting in the life sciences. Proc Natl Acad Sci. 2021 Apr 27;118(17):e2103238118. doi: 10.1073/pnas.2103238118 33893240 PMC8092464

[33] Juster R-P, Perna A, Marin M-F, Sindi S, Lupien SJ. Timing is everything: Anticipatory stress dynamics among cortisol and blood pressure reactivity and recovery in healthy adults. Stress. 2012 Nov;15(6):569–577. doi: 10.3109/10253890.2012.661494 22296506

[34] Longpré C, Lupien SJ. Effect of positive news media on stress reactivity, memory and affect in young adults. DATASET [Internet]. 2021. doi: 10.17605/OSF.IO/4ZX98 PMC8553098 34710138

[35] SPSS Statistics [Internet]. United States: IBM; 2021 [cited 2021 May 31]. https://www.ibm.com/products/spss-statistics

[36] R: A language and environment for statistical computing. R Foundation for Statistical Computing, [Internet]. Vienna, Austria; 2020. https://www.R-project.org/.

[37] Kirschbaum C, Kudielka BM, Gaab J, Schommer NC, Hellhammer DH. Impact of Gender, Menstrual Cycle Phase, and Oral Contraceptives on the Activity of the Hypothalamus-Pituitary-Adrenal Axis: Psychosom Med. 1999;61(2):154–162. doi: 10.1097/00006842-199903000-00006 10204967

[38] Pituch KA, Stevens JP. Applied multivariate statistics for the social sciences: analyses with SAS and IBM’s SPSS. Sixth edition. New York London: Routledge, Taylor and Francis Group; 2016. 793 p.

[39] Aczel B, Palfi B, Szaszi B. Estimating the evidential value of significant results in psychological science. Wicherts JM, editor. PLOS One. 2017 Aug 18;12(8):e0182651. doi: 10.1371/journal.pone.0182651 28820905 PMC5562314

[40] Aczel B, Palfi B, Szollosi A, Kovacs M, Szaszi B, Szecsi P, et al. Quantifying Support for the Null Hypothesis in Psychology: An Empirical Investigation. Adv Methods Pract Psychol Sci. 2018 Sep;1(3):357–366.

[41] Wagenmakers E-J. JASP [Internet]. Amsterdam; 2021 [cited 2021 May 31]. https://jasp-stats.org/

[42] Jarosz AF, Wiley J. What Are the Odds? A Practical Guide to Computing and Reporting Bayes Factors. J Probl Solving [Internet]. 2014 Nov 7 [cited 2020 Sep 17];7(1). Available from: https://docs.lib.purdue.edu/jps/vol7/iss1/2

[43] Lewicka M, Czapinski J, Peeters G. Positive-negative asymmetry or ‘When the heart needs a reason’. Eur J Soc Psychol. 1992;22(5):425–434.

[44] Shoemaker PJ. Hardwired for News: Using Biological and Cultural Evolution to Explain the Surveillance Function. J Commun. 1996;46(3):32–47.

[45] Ito TA, Larsen JT, Smith NK, Cacioppo JT. Negative information weighs more heavily on the brain: The negativity bias in evaluative categorizations. J Pers Soc Psychol. 1998;75(4):887–901. doi: 10.1037//0022-3514.75.4.887 9825526

[46] Baumeister RF, Bratslavsky E, Finkenauer C, Vohs KD. Bad is Stronger than Good. Rev Gen Psychol. 2001 Dec 1;5(4):323–370.

[47] Rozin P, Royzman EB. Negativity Bias, Negativity Dominance, and Contagion. Personal Soc Psychol Rev. 2001 Nov 1;5(4):296–320.

[48] Zillmann D, Chen L, Knobloch S, Callison C. Effects of Lead Framing on Selective Exposure to Internet News Reports. Commun Res. 2004 Feb;31(1):58–81.

[49] Soroka S, McAdams S. News, Politics, and Negativity. Polit Commun. 2015 Jan 2;32(1):1–22.

[50] Knobloch-Westerwick S, Mothes C, Polavin N. Confirmation Bias, Ingroup Bias, and Negativity Bias in Selective Exposure to Political Information. Commun Res. 2020 Feb 1;47(1):104–124.

[51] Soroka S, Fournier P, Nir L. Cross-national evidence of a negativity bias in psychophysiological reactions to news. Proc Natl Acad Sci. 2019 Sep 17;116(38):18888–18892. doi: 10.1073/pnas.1908369116 31481621 PMC6754543

[52] Poppenk J, Köhler S, Moscovitch M. Revisiting the novelty effect: When familiarity, not novelty, enhances memory. J Exp Psychol Learn Mem Cogn. 2010;36(5):1321–1330. doi: 10.1037/a0019900 20804299

[53] Bell A. News Time. Time Soc. 1995 Oct 1;4(3):305–328.

[54] Hellhammer J, Schubert M. The physiological response to Trier Social Stress Test relates to subjective measures of stress during but not before or after the test. Psychoneuroendocrinology. 2012 Jan;37(1):119–124. doi: 10.1016/j.psyneuen.2011.05.012 21689890

[55] Anderson SF. Misinterpreting p: The discrepancy between p values and the probability the null hypothesis is true, the influence of multiple testing, and implications for the replication crisis. Psychol Methods. 2020;25(5):596–609. doi: 10.1037/met0000248 31829657

