# Supplementary material for: Staying informed without a cost: No effect of positive news media on stress reactivity, memory and affect in young adults
Source: PLoS One. 2021 Oct 28;16(10):e0259094. doi: 10.1371/journal.pone.0259094 (PMC8553098; doi:10.1371/journal.pone.0259094)
Supplement: S1 Table — 12 neutral news and 12 positive news segments used as stimuli in the present study. The items have been translated into English for the purpose of this article, but the original language of the news is French. (PDF) [file pone.0259094.s001.pdf]

S1 Table

News segments (neutral news and positive news) used in the current study. 12 neutral news and 12 positive news segments used as stimuli in the present study. The items have been translated into English for the purpose of this article, but the original language of the news is French.

**Neutral news segments**	
**Original item**	**Translated item**
2Frères offert en streaming. Le duo 2Frères, qui a vendu 170 000 albums depuis le début de sa carrière en 2015, a annoncé cette semaine que ses deux disques seraient dorénavant offerts en *streaming*.	2Frères available for streaming. The 2Frères duo, who have sold 170,000 albums since the start of their career in 2015, announced this week that both of their records would now be able for streaming.
Un duplex converti en unifamiliale à Verdun. Cet ancien duplex a été converti en unifamiliale par l'entrepreneure Marie-Jeanne Rivard dans le cadre de la première saison de l'émission Flip de fille.	A duplex converted into a single-family home in Verdun. This old duplex was converted into a single-family home by the entrepreneur Marie-Jeanne Rivard for the first season of the show Flip de fille.
Apple renonce à son chargeur sans fil annoncé en 2017. Fait rare, Apple a officiellement renoncé à lancer un produit, en l'occurrence un chargeur sans fil baptisé AirPower promis depuis 2017, évoquant l'impossibilité de mettre au point un appareil digne de la firme à la pomme.	Apple abandons its wireless charger announced in 2017. In a rare instance, Apple has officially abandoned the launch of a product, in this case a wireless charger called AirPower that was promised since 2017, citing the impossibility of developing a device worthy of the Apple firm.
Hubert Lenoir se rase les cheveux dans le clip Fille de personne II. La chanson a beau tourner en boucle depuis plus d'un an, Hubert Lenoir a lancé le clip officiel de Fille de personne II. On peut y voir le chanteur originaire de Beauport en tournée et sur scène, en France et au Québec, mener sa vie d'artiste pop à fond de train avec son équipe.	Hubert Lenoir shaves his head in the video clip Fille de personne II. The song may have been playing on a loop for over a year, but Hubert Lenoir has released the official video for Fille de personne II. It shows the Beauport-born singer on tour and on stage in France and Quebec, living his life as a pop star with his team at full speed.
Le Chalet : La fin du Friends québécois. Le dernier épisode de la série Le Chalet a été diffusé sur les ondes de Vrak. Après cinq saisons à suivre les péripéties de cette bande de six copains, il faudra dire adieu à Sarah, Antoine, Charles, Lili, Frank et Cath. Retour sur le phénomène Le Chalet.	Le Chalet: The end of the Quebec Friends. The last episode of the series The Chalet was broadcoasted on Vrak. Ater five seasons of following the adventures of this group of six friends, we will have to say farewell to Sarah, Antoine, Charles, Lili, Frank, and Cath. A look back at The Chalet experience.
Les chats reconnaissent leur nom. Les chercheurs nippons ont visité des familles où habitaient un nombre variable de chats, ainsi qu'un café où vivent des chats. Ils ont appelé les chats par leur	Cats recognize their names. Japanese researchers visited families with varying numbers of cats as well as a coffee shop where cats live. They called the cats by name. Each time, a characteristic

S1 Table

nom. À chaque fois, un mouvement caractéristique de la tête et des oreilles a eu lieu, mouvement qui ne se produisait pas quand d'autres mots étaient prononcés.	movement of the head and ears occurred, which did not occur when other words were said.
Dans l'ombre de Stroll, Latifi connaît un bon début de saison en F2. Dans l'ombre de Lance Stroll, le Canadien Nicholas Latifi est conscient qu'il doit tourner pour s'améliorer. Latifi doit développer sa constance, et pour y parvenir il doit tourner le plus possible sur un circuit qui se veut l'antichambre de la F1.	In the shadow of Stroll, Latifi is having a good start to the F2 season. In the shadow of Lance Stroll, Canadian Nicholas Latifi is aware that he needs to race to improve. Latifi needs to develop consistency, and to do so, he needs to race as much as possible on a circuit that resembles that of the F1.
Maintien du taux directeur à 1,75 % au Canada. La Banque du Canada annonce le maintien de son taux d'intérêt directeur à 1,75 %.	Policy rate maintained at 1.75% in Canada. The Bank of Canada announces that its key policy rate will remain at 1.75%.
Benoît Brière met sur le marché sa majestueuse résidence. Le comédien et metteur en scène Benoît Brière a mis en vente sa majestueuse résidence située dans le quartier parc Victoria à Saint-Lambert, sur la Rive-Sud de Montréal.	Benoît Brière puts his majestic residence on the market. The actor and director Benoît Brière has put his majestic residence up for sale in the Victoria Park neighborhood of Saint-Lambert on the south shore of Montreal.
Patinage de vitesse : la retraite pour Keri Morrison. La patineuse de vitesse Keri Morrison, qui a représenté le Canada autant en courte piste qu'en longue piste, a officiellement pris sa retraite, a annoncé Patinage de vitesse Canada par voie de communiqué.	Speed skating: retirement for Keri Morrison. Speed Skating Canada annouced in a press release that speed skater Keri Morrison, who represented Canada in both short and long track, has officially retired.
Le Walkman souffle ses 40 bougies. Le 1er juillet 1979, le premier Walkman de Sony, le TPS-L2, était lancé sur le marché. 40 ans plus tard, bien que le temps d'écouter de la musique sur cassette soit révolu, l'idée de transporter sa musique a fait bien du chemin.	The Walkman celebrates its 40th birthday. On July 1st 1979, the first Walkman by Sony, the TPS-L2, was released on the market. Fourty years later, although the days of listening to music on tape are over, the idea of carrying music on our person has come a long way.
Un nouveau sergent d'armes est nommé à Ottawa. Le premier ministre Justin Trudeau a salué la nomination de Patrick McDonell au poste de sergent d'armes officiel de la Chambre des communes. M. McDonell occupe le poste de sergent d'armes par intérim et de chef de la sécurité à la Chambre des communes depuis 2015.	A new sergeant-at-arms is appointed in Ottawa. Prime minister Justin Trudeau has welcomed the appointment of Patrick McDonell as the official sergeant-at-arms of the House of Commons. Mr. McDonell has served as acting sergeant-at-arms and chief of security at the House of Commons since 2015.

S1 Table

Positive news segments	
**Original item**	**Translated item**
Cet enfant de 12 ans offre des milliers de repas aux sans-abris. Liam Hannon, 12 ans, a fondé une organisation « Liam's Lunches of Love » consacrée à la distribution de repas aux sans-abris. Il a déjà offert plus de 2000 déjeuners aux sans-abris de son quartier.	A 12-year-old child provides thousands of meals to the homeless. Liam Hannon, 12 years of age, founded an organization called "Liam's Lunches of Love" dedicated to providing meals to the homeless. He has already provided more than 2000 lunches to the homeless in his neighborhood.
Bientôt des gouttes pour guérir la cataracte? Des scientifiques viennent de mettre au point un traitement médicamenteux qui pourrait complètement guérir les personnes atteintes de cataracte.	Will we soon have eye drops to cure cataracts? Scientists have just developed a drug treatment that could completely cure people with cataracts.
Montréal : Un hôpital accueille des sans-abris pour l'hiver. L'hôpital historique de Montréal, le Royal Victoria fermé depuis 2015, a été transformé en un refuge temporaire pour sans-abri, afin d'assurer que personne ne dorme dans le froid hivernal.	Montreal: A hospital welcomes the homeless for the winter. Montreal's historic Royal Victoria hospital, closed since 2015, has been transformed into a temporary homeless shelter to ensure no one sleeps in the winter cold.
Une chaîne humaine de 620km pour lutter contre le sexisme. Ce ne sont pas moins de 5 millions d'Indiennes qui se sont réunies le 1er janvier 2019 afin de constituer un véritable « mur de femmes ». Le but principal de cette chaîne humaine ? La lutte pour les droits des femmes et contre le sexisme et le patriarcat en Inde.	A 620 km human chain to fight against sexism. No less than five million Indian women gathered on January 1st 2019 to form a "wall of women". The main goal of this human chain? To fight for women's rights, against sexism, and patriarchy in India.
Finlande : Un programme contre le harcèlement scolaire. Un programme de lutte contre l'intimidation à l'école a été mis en place dans 75 % des établissements scolaires du pays, pour les élèves de 6 à 16 ans. Cette méthode a diminué le harcèlement scolaire de 85 %.	Finland: An anti-bullying program for schools. An anti-bullying school program has been implemented in 75% of the country's schools for students aged 6 to 16. This method has reduced bullying in schools by 85%.
La couche d'ozone est en train de se reconstituer. Selon les Nations Unies, la couche d'ozone se remet peu à peu des dommages créés par les produits chimiques. Le rapport précise que les trous dans la couche d'ozone au-dessus de la zone antarctique se réduisent peu à peu, chaque année.	The ozone layer is recovering. According to the United Nations, the ozone layer is slowly recovering from the damage created by chemical products. The report states that the holes in the ozone layer over the Antarctic are slowly shrinking each year.
Un aspirateur de CO2 géant au Canada. Une équipe de chercheurs de l'Université de l'Alberta	A giant CO2 vacuum cleaner in Canada. A team of researchers at the University of Alberta in Calgary

S1 Table

à Calgary a mis au point une machine capable de capturer le dioxyde de carbone présent dans l'air.	have developed a machine capable of capturing carbon dioxide present in the air.
Un nouveau vaccin capable de neutraliser le virus Ebola? Selon une étude récente, l'anticorps identifié par des chercheurs américains serait capable de neutraliser les trois souches du virus Ebola qui touchent l'être humain.	A new vaccine capable of neutralizing the Ebola virus? According to a recent study, the antibody identified by American researchers would be able to neutralize the three strains of the Ebola virus that affect humans.
Montréal : Bientôt un vaccin contre le cancer? Cela fait 40 ans que le Dr Claude Perreault s'est engagé dans une quête de nouveaux traitements contre le cancer. Il essaie actuellement de concevoir des vaccins capables de guérir le cancer. Il espère pouvoir tester ces vaccins thérapeutiques sur des humains d'ici 3 ans.	Montreal: A cancer vaccine coming soon? For 40 years, Dr. Claude Perreault has been on a quest to find new cancer treatments. He is currently trying to develop vaccines that cure cancer. He hopes to be able to test these therapeutic vaccines on humans within the next three years.
Turquie : Une bouteille en plastique contre un ticket de métro. La ville d'Istanbul a mis en place un moyen original pour que les usagers manquant d'argent puissent se procurer un ticket de transport en échangeant des matières recyclables contre des crédits, afin de pouvoir se déplacer dans la ville.	Turkey: A plastic bottle for a metro ticket. The city of Istanbul has established an original way for users without money to get a transportation ticket by exchanging recyclable materials for funds, in order to get around the city.
Angleterre : Elle empêche des suicides grâce à des messages d'espoirs. Elle écrit des messages simples, mais encourageants et positifs sur des petites feuilles colorées, pour les accrocher sur le pont Wearmouth, un pont réputé pour les nombreux suicides qui y ont eu lieu. Depuis l'affichage des notes sur le pont, la jeune femme a sauvé la vie d'au moins huit personnes.	England: She prevents suicide with messages of hope: She writes simple, but encouraging and positive messages on small coloured sheets, to hang on the Wearmouth Bridge, a bridge known for the many suicides that have taken place there. Since posting the notes on the bridge, the young woman has saved the lives of at least eight people.
Montréal : ces souris pourraient soigner le cancer. Soigner les maladies comme l'obésité, le diabète et même le cancer semble maintenant envisageable grâce à une découverte concernant les souches de souris de laboratoire. Des chercheuses du centre de recherche Maisonneuve-Rosemont ont participé à cette étude.	Montreal: these mice could cure cancer. Treating diseases like obesity, diabetes, and even cancer now seems possible thanks to a discovery involving laboratory mice strains. Researchers from the Maisonneuve-Rosemont research centre participated in this study.
